# Brain Fatty Acid Composition and Inflammation in Mice Fed with High-Carbohydrate Diet or High-Fat Diet

**DOI:** 10.3390/nu10091277

**Published:** 2018-09-10

**Authors:** Lorena Gimenez da Silva-Santi, Marina Masetto Antunes, Marco Aurélio Mori, Camila Biesdorf de Almeida-Souza, Jesuí Vergílio Visentainer, Fabiana Carbonera, Amanda Rabello Crisma, Laureane Nunes Masi, Sandro Massao Hirabara, Rui Curi, Roberto Barbosa Bazotte

**Affiliations:** 1 Department of Pharmacology and Therapeutics, State University of Maringá, Maringá 87020-900, Paraná, Brazil; lorenajufem@gmail.com (L.G.d.S.-S.); marinaantunes_1992@hotmail.com (M.M.A.); marcoaureliomori@gmail.com (M.A.M.); camila.biesdorf1@gmail.com (C.B.d.A.-S.); 2 Department of Chemistry, State University of Maringá, Maringá 87020-900, Paraná, Brazil; jesuiv@gmail.com (J.V.V.); fabianacarbonera@gmail.com (F.C.); 3 Department of Clinical Analysis, Federal University of Paraná, Curitiba 80210-170, Paraná, Brazil; amycrisma@yahoo.com.br; 4 Interdisciplinary Post-Graduate Program in Health Sciences, Cruzeiro do Sul University, São Paulo 03342-000, Brazil; laure_masi@hotmail.com (L.N.M.); sandromh@yahoo.com.br (S.M.H.); curirui@gmail.com (R.C.)

**Keywords:** saturated fatty acids, monounsaturated fatty acids, polyunsaturated fatty acids, *n*-6/*n*-3 fatty acid ratio, cytokines, nutrients

## Abstract

Both high fat diet (HFD) and high carbohydrate diet (HCD) modulate brain fatty acids (FA) composition. Notwithstanding, there is a lack of information on time sequence of brain FA deposition either for HFD or HCD. The changes in brain FA composition in mice fed with HFD or HCD for 7, 14, 28, or 56 days were compared with results of 0 (before starting given the diets). mRNA expressions of allograft inflammatory factor 1 (Aif1), cyclooxygenase-2 (Cox 2), F4/80, inducible nitric oxide synthase (iNOS), integrin subunit alpha m (Itgam), interleukin IL-1β (IL-1β), IL-6, IL-10, and tumor necrosis factor alpha (TNF-α) were measured. The HFD group had higher speed of deposition of saturated FA (SFA), monounsaturated FA (MUFA), and polyunsaturated FA (PUFA) at the beginning of the experimental period. However, on day 56, the total amount of SFA, MUFA, and PUFA were similar. mRNA expressions of F4/80 and Itgam, markers of microglia infiltration, were increased (*p* < 0.05) in the brain of the HCD group whereas inflammatory marker index (IMI) was higher (46%) in HFD group. In conclusion, the proportion of fat and carbohydrates in the diet modulates the speed deposition of FA and expression of inflammatory gene markers.

## 1. Introduction

Diet has been associated with brain function and alteration in of diet composition has been considered as a risk factor for the development of brain diseases [1,2,3,4,5]. 

Lipid content represents more than half of brain dry weight and has a pivotal role for its integrity and function. For example, polyunsaturated FA (PUFA) such as arachidonic acid (AA, 20:4*n*-6), docosahexaenoic acid (DHA, 22:6*n*-3), eicosapentaenoic acid (EPA, 20:5*n*-3), and γ-linolenic acid (18:3*n*-6) are structural and functional component of the brain. A high content of saturated fatty acids (SFA) reduces the flexibility and elasticity of nerve cells [6,7,8]. Neuroinflammation is a hallmark of brain diseases and it has been associated with the FA deposition [9,10,11]. 

In a previous study, both high-carbohydrate diet (HCD) and high-fat diet (HFD) modulated FA accumulation and inflammation in the liver [12]. The time sequence of changes induced by both diets on brain FA composition has not yet been determined and compared yet.

In this investigation, the time course of changes in brain FA composition—induced by HFD or HCD for 0 (before starting the diets), 7, 14, 28, or 56 days—were evaluated and compared with results on day 0 (before starting the diets). In addition, mRNA expressions of allograft inflammatory factor 1 (Aif1), cyclooxygenase-2 (Cox 2), F4/80, integrin subunit alpha m (Itgam), interleukin IL-1β (IL-1β), IL-6, IL-10, and tumor necrosis factor alpha (TNF-α) were measured.

## 2. Materials and Methods 

### 2.1. Animals and Treatments

Male Swiss mice (total = 72 animals) were maintained in standard laboratory conditions in a photoperiod (12 h light/12 h darkness), temperature (22 ± 1 °C), and humidity-controlled environment. Food and water were available ad libitum. 

All experiments were carried out in accordance with the international guidelines for the use and care of laboratory animals approved by the Scientific Advisory Committee on Animal Care of State University of Maringá (protocol 002/2014).

Mice (six weeks of age) were fed with standard rodent chow (Nuvilab^®^, Curitiba, PR, Brazil) before the initiation of the experimental protocol. 

After three days of acclimatization, the animals (weighing about 35 g) were divided into two groups: HFD and HCD. 

The amounts of protein, carbohydrate, and total fat were 20.3, 36.5, and 35.2 g/100 g for the high fat diet, and 14.2, 73.8, and 4.0 g/100 g for the high carbohydrate diet, respectively. Highly refined ingredients (Rhoster Company, Araçoiaba da Serra, SP, Brazil) were used to prepare diets. The diets composition were based on purified diets for maintenance of laboratory adult rodents proposed by the American Institute of Nutrition (AIN-93-M) [13]. Details about the composition of the diets can be found in our previous work [12].

Mice fed with HFD or HCD for 0 (before starting the diets), 7, 14, 28, or 56 days (*n* = 8 for each time of treatment with HCD or HFD) were fasted (from 5:00 p.m. to 8:00 a.m.), before being sacrifice by decapitation. 

The brains were quickly and carefully removed immediately prior to the liver that was used in our previous study [12], frozen in liquid nitrogen, and stored at −80 °C until analysis being performed. 

### 2.2. Fatty Acid Composition Analysis

A method in reduced scale was used to extract the total lipid content of the brain samples. For this purpose, 1.000 ± 0.001 g of homogenized brain samples were used. FA methyl esters (FAME) of brain homogenates were prepared by ultrasound to assist total lipid methylation as described by Santos et al. [14]. FAME separation was performed by gas chromatography in a Thermo Scientific™TRACE™Ultra Gas Chromatographer (Thermo Scientific™, Waltham, MA, USA), fitted with a flame ionization detector (FID) and a fused-silica capillary column. For identification of the FAs, the retention times were compared to those of standard methyl esters. The results of FA contents in the brain were expressed as mg/100 mg sample. More details about this methodology can be found in our previous study [12]. 

### 2.3. Expressions of Inflammatory Genes and Estimation of the Inflammatory Marker Index (IMI)

Total RNA was extracted using TRIzol reagent (Invitrogen Life Technologies, Waltham, MA, USA) and reverse transcribed to cDNA (High-Capacity cDNA kit, Applied Biosystems, Foster City, CA, USA). Gene expression was evaluated by real-time PCR using SYBR Green as the fluorescent dye (Invitrogen Life Technologies, Waltham, MA, USA). Primer sequences are in Table 1. Analysis of gene expression was performed according to a previously described method [15], using ribosomal protein lateral stalk subunit P0 gene (Rplp0) as the internal control.

The IMI was calculated by the sum of expressions of F4/80 + IL-6 + IL-1β + TNFα + iNOS + COX-2 + Itgam + Aif1 (pro-inflammatory factors) divided by IL-10 (anti-inflammatory factor), as previously described [12].

### 2.4. Statistical Analysis 

Results are reported as the mean ± standard deviation of the mean and analyzed by Student’s *t*-test or ANOVA, followed by the post-test of Tukey using the Graph-Pad Prism Version 5.0 software (Graph Pad Software Inc., San Diego, CA, USA) to assess differences between means. *p*-values < 0.05 were used to indicate statistical significances.

## 3. Results

### 3.1. Brain Fatty Acid Deposition

Brains from the HFD and HCD groups had higher content of palmitic acid (16:0; Figure 1), stearic acid (18:0; Figure 1), oleic acid (18:1*n*-9; Figure 2), AA (20:4*n*-6; Figure 3), and DHA (22:6*n*-3; Figure 4), as compared with other FA. 

**Figure 1:**
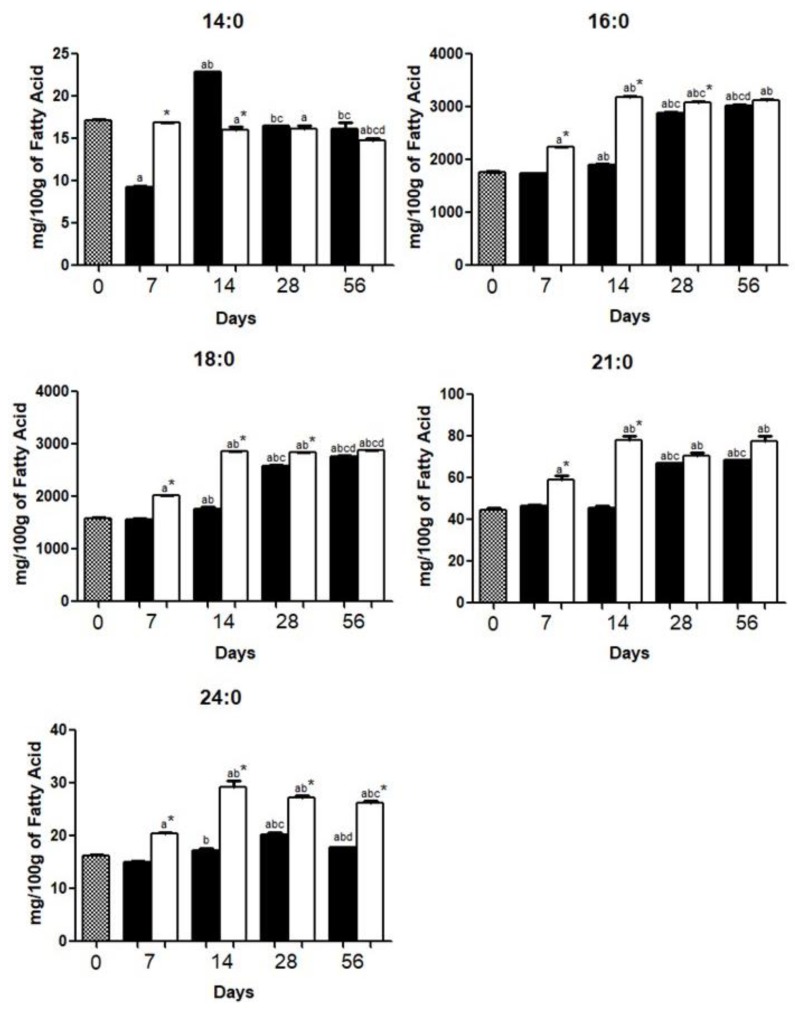
Saturated fatty acid (SFA) composition in the brains of mice fed a high carbohydrate diet (HCD) or high fat diet (HFD) before (day 0) and 7, 14, 28, or 56 days after onset of diet administration. The concentrations of myristic acid (**14:0**), palmitic acid (**16:0**), stearic acid (**18:0**), heneicosanoic acid (**21:0**), and tetracosanoic acid (**24:0**) were expressed as the mean ± standard deviation of three replicates for each group. *p* < 0.05 as compared with day 0 (**a**), day 7 (**b**), day 14 (**c**), and day 28 (**d**), and HCD group*. 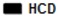 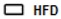 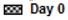.

We observed an increase (*p* < 0.05) in palmitic acid (16:0), stearic acid (18:0), heneicosanoic acid (21:0), and tetracosanoic acid (24:0) content between day 0 and day 56, in both HCD and HFD groups. Except for tetracosanoic acid (24:0), the brain of the HCD or HFD group exhibited a similar SFA content on day 56 (Figure 1).

**Figure 2:**
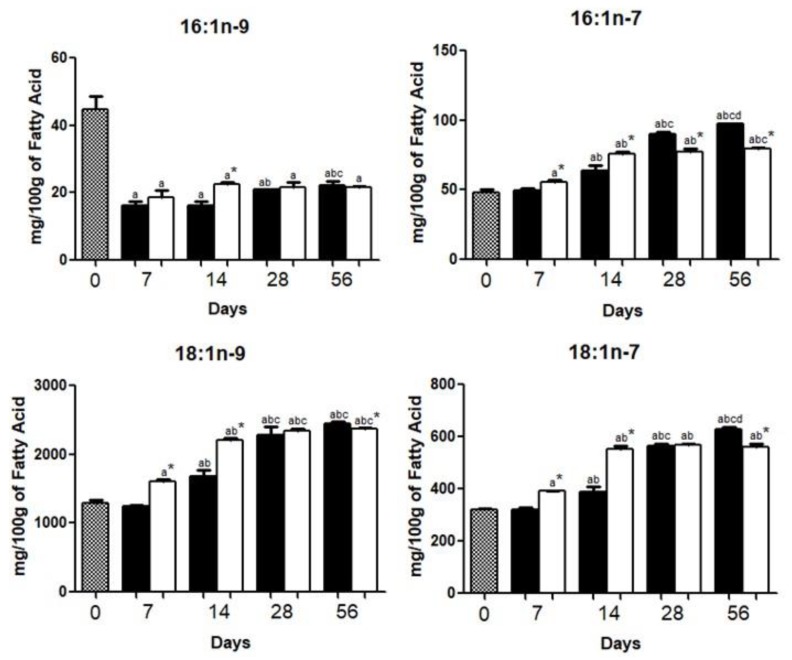
Monounsaturated fatty acid (MUFA) composition in the brains of mice fed a high carbohydrate diet (HCD) or high fat diet (HFD) before (day 0) and 7, 14, 28, or 56 days after onset of diet administration. The concentrations of 7-hexadecanoic acid (**16:1*n*-9**), palmitoleic acid (**16:1*n*-7**), oleic acid (**18:1*n*-9**), and vaccenic acid (**18:1*n*-7**) were expressed as the mean ± standard deviation of three replicates for each group. *p* < 0.05 as compared with day 0 (**a**), day 7 (**b**), day 14 (**c**), and day 28 (**d**), and HCD group*. 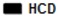 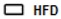 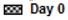.

The content of 7-hexadecanoic acid (16:1*n*-9) was decreased (*p* < 0.05) from day 7 until day 56 in the brains of both groups. Brains of the HFD group had lower (*p* < 0.05) palmitoleic acid (16:1*n*-7), oleic acid (18:1*n*-9), and vaccenic acid (18:1*n*-7) after 56 days (Figure 2). 

**Figure 3:**
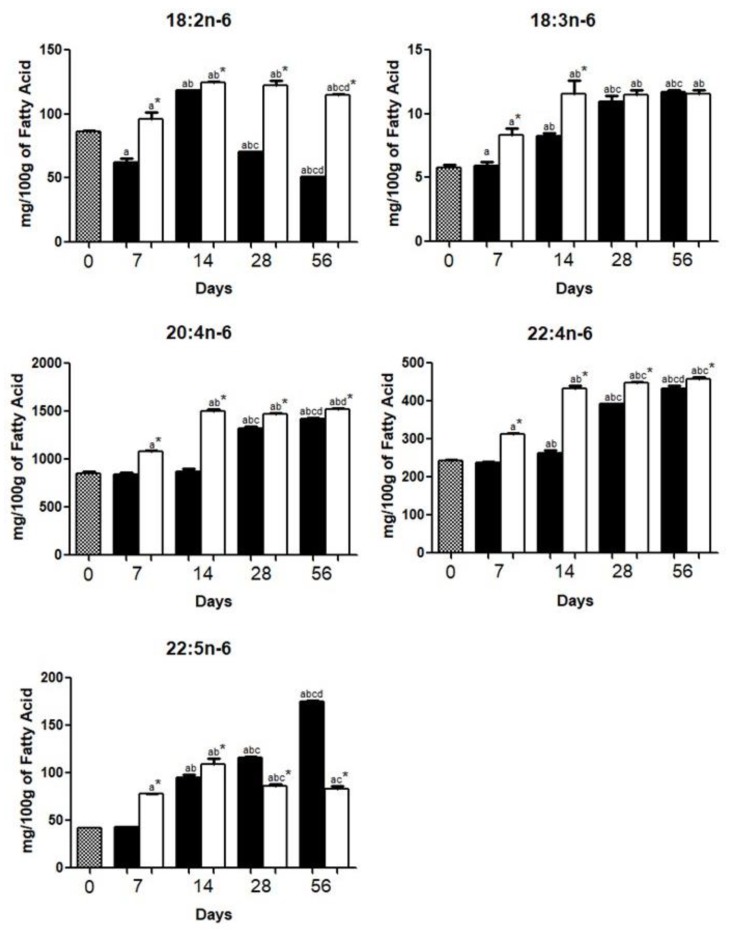
Polyunsaturated *n*-6 fatty acid (*n*-6 PUFA) composition in the brains of mice fed a high carbohydrate diet (HCD) or high fat diet (HFD) before (day 0) and 7, 14, 28, or 56 days after onset of diet administration. The levels of linoleic acid (**18:2*n*-6**), γ-linolenic acid (**18:3*n*-6**), arachidonic acid (**20:4*n*-6**), docosatetraenoic acid (**22:4*n*-6**), and docosapentaenoic acid (**22:5*n*-6**) were expressed as the mean ± standard deviation of three replicates for each group. *p* < 0.05 as compared with day 0 (**a**), day 7 (**b**), day 14 (**c**), and day 28 (**d**), and HCD group*. 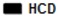 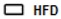 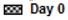.

The content of linoleic acid (18:2*n*-6) was decreased (*p* < 0.05) and increased (*p* < 0.05) in the brain of HCD and HFD, respectively (Figure 3). 

γ-linolenic acid (18:3*n*-6), AA (20:4*n*-6), docosatetraenoic acid (22:4*n*-6), and docosapentaenoic acid (22:5*n*-6) were increased between day 0 and day 56 (HFD or HCD). The increase was more pronounced for brain docosapentaenoic acid (22:5*n*-6) content from day 56 in HCD compared to HFD (Figure 3). 

**Figure 4:**
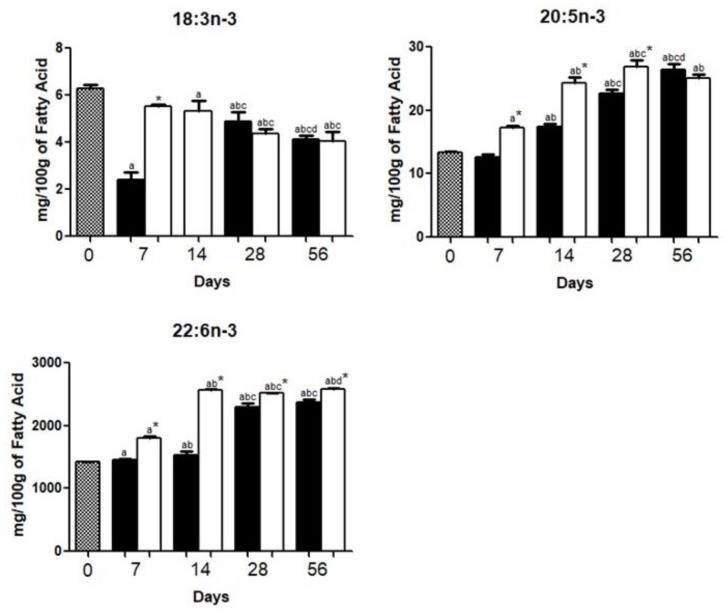
Polyunsaturated *n*-3 fatty acid (*n*-3 PUFA) composition in the brains of mice fed a high carbohydrate diet (HCD) or high fat diet (HFD) before (day 0) and 7, 14, 28, or 56 days after onset of diet administration. The contents of α-linolenic acid (**18:3*n*-3**), eicosapentaenoic acid (EPA, **20:5*n*-3**), docosahexaenoic acid (DHA, **22:6*n*-3**) were expressed as the mean ± standard deviation of three replicates for each group. *p* < 0.05 as compared with day 0 (**a**), day 7 (**b**), day 14 (**c**), and day 28 (**d**), and HCD mice*. 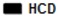 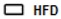 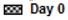.

EPA (20:5*n*-3) and DHA (22:6*n*-3) levels were increased (*p* < 0.05) whereas α-linolenic acid (ALA, 18:3*n*-3) levels were decreased (*p* < 0.05) during the 56-day period, either for HFD or HCD (Figure 4). 

SFA represents approximately 45% of the total brain FA, either in HCD or HFD mice whereas MUFA represents approximately 25% of the total brain FA, either in HCD or HFD (Table 2).

PUFA represent approximately 35% of the total brain FA, being n-3 PUFA a half of this percentage, either in HCD or HFD (Table 2).

The brains exhibited similar PUFA/SFA, MUFA/SFA, and n-6/n-3 ratio throughout the 56-day period regardless of diet given (Table 2). 

Lipid accumulation, calculated by the sum of all FA of each family (SFA, MUFA, and PUFA) was faster in the brains of HFD mice. The HFD group reached the maximum FA accumulation on day 28, whereas HCD mice reached maximum value on day 56 only. After 56 days, however, the sum of all FA evaluated, i.e., SFA plus MUFA plus PUFA (HFD group vs. HCD group) was similar (Table 2).

### 3.2. Expression of Inflammatory Genes

Brains from the HFD group exhibited lower (*p* < 0.05) mRNA expressions of the F4/80 and Itgam (Table 3) on day 56. The IMI was higher (46%) in the brain of HFD mice. 

## 4. Discussion

### 4.1. Brain Fatty Acid Deposition

Brain FA profile is tightly regulated and exhibits only a lower response to diet composition changes in comparison with other tissues like liver, skeletal muscle, and heart [16,17,18,19]. 

In agreement with other studies [20,21], a predominance of palmitic acid (16:0), stearic acid (18:0), oleic acid (18:1*n*-9), AA (20:4*n*-6), and DHA (22:6*n*-3) was described in the brains of both groups during the experimental period. 

SFA can activate transcription factors of glial cells and the innate immune system, triggering expression of pro-inflammatory genes such as cytokines, chemokines, iNOS, and COX. SFA also activates the nuclear factor-kappa B (NF-κB) that raises the expressions of inflammatory genes [22]. As a consequence, the inflammatory state is induced in the neurons by these fatty acids [23,24]. 

As we previously quantified [12] the amount of SFA in the high fat diet is more than five times higher than that found in the high carbohydrate diet. Nevertheless, except for tetracosanoic acid (24:0), either HFD or HCD mice exhibited similar brain SFA composition on day 56 (Figure 1). The tetracosanoic acid (24:0), found in higher quantities in brains of the HFD group is reported to be toxic for oligodendrocytes and astrocytes [25]. Reduced myristic acid content (14:0) was reported either in HFD or HCD mice between day 0 and day 56. This FA is an important cellular component of several proteins that require myristoylation to exert their biological effects [26].

The amount of MUFA in the HFD is more than five times higher if compared with HCD [12]. Notwithstanding, except for 7-hexadecanoic acid (16:1*n*-9), HFD and HCD mice exhibited similar brain MUFA composition on day 56.

Oleic acid (18:1*n*-9) was increased (*p* < 0.05) in both groups at day 56, being more pronounced in HCD. This FA has anti-inflammatory properties in the brain by inhibiting activation of NF-kB signaling pathways in neurons and astrocytes. Oleic acid also promotes axonogenesis in the striatum during brain development and it is used as a brain energy source during the decreased availability of glucose [27,28].

ALA (18:3*n*-3) and LA (18:2*n*-6), the main precursors of PUFA in the brain may be converted into DHA (22:6*n*-3) and AA (20:4*n*-6), respectively, by desaturation and elongation. *n*-3 PUFA and *n*-6 PUFA compete for the same desaturases and elongases [29]. Since *n*-6/*n*-3 ratios in the diets are similar [12], the competition for these enzymes is probably equivalent for both groups.

Brain LA (18:2*n*-6), was increased (*p* < 0.05) and decreased (*p* < 0.05) in HFD and HCD, respectively. This difference might be explained by the fact that HFD mice had a higher (*p* < 0.05) content of palmitic acid (16:0) in the diet [12] and the entry of LA (18:2*n*-6) into the brain is proportional to the blood concentration of palmitic acid (16:0) [30]. 

Circulating ALA (18:3*n*-3) and LA (18:2*n*-6) are able to cross the blood-brain barrier being then converted into DHA (22:6*n*-3) and AA (20:4*n*-6) in the brain [31]. ALA (18:3*n*-3) and LA (18:2*n*-6) from the diet can also be converted to DHA (22:6*n*-3) and AA (20:4*n*-6) in the liver and then released to bloodstream [24]. DHA is taken up by the brain in preference to other fatty acids [32]. 

Brain is rich in PUFA, which represent about 20% of the brain dry weight [29]. PUFA levels in this study agree with previous reports in which DHA (22:6*n*-3) and AA (20:4*n*-6) are the main *n*-3 and *n*-6 PUFA, respectively [33,34]. Between day 0 and day 56 period, there was an increase (*p* < 0.05) of DHA and AA in brains of either HCD or HFD mice (Figure 3 and Figure 4).

EPA (20:5*n*-3) content was only about 1% of that of DHA (22:6n-3). Brain EPA (20:5*n*-3) content is very low probably because of its intense metabolism. This FA is rapidly converted through β-oxidation, elongation, and desaturation to docosapentaenoic acid (22:5*n*-3) and DHA (22:6*n*-3) [35].

HCD mice had a more intense increase (*p* < 0.05) of docosapentaenoic acid (22:5*n*-6) content, a metabolite of LA (18:2*n*-6). Ghosh et al. [36] reported a reduction of this FA in cell membranes of prefrontal white matter in postmortem patients with bipolar disorder and schizophrenia.

The content of the docosatetraenoic acid (22:4*n*-6), a product of elongase activity [37], was increased (*p* < 0.05) in the brain of the HFD and HCD groups, respectively. 

PUFA, which are abundant in cell membrane phospholipids of neural tissues [38], have a pivotal role for maintaining membrane fluidity, permeability, lipid–protein and lipid–lipid interactions for brain neurogenesis and modulation of inflammation [37,39]. 

The pro-inflammatory mediators derived from AA (20:4*n*-6)—i.e., prostaglandins, thromboxanes, leukotrienes, and lipoxins—intensify neuroinflammation [11]. In contrast, DHA (22:6*n*-3) and EPA (20:5*n*-3) have anti-inflammatory, antiapoptotic, and antioxidant properties [40].

**Figure 5:**
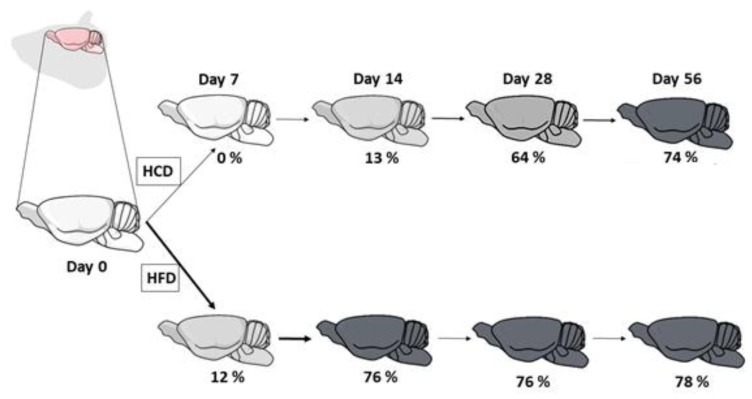
Summary of the results in brains of mice fed with either high carbohydrate diet (HCD) or high fat diet (HFD). The grey color indicates the intensity of fatty acid (FA) accumulation (%). Bold arrows indicate faster FA deposition. HFD exhibited an increased speed of FA deposition, however, at the end of 56 days, HFD and HCD had the same amount of brain FA content.

HFD mice had a faster brain deposition of FA, reaching the maximum FA accumulation on day 14 whereas HCD reached maximum FA deposition on day 14 or 28. However, on day 56, the sum of SFA, MUFA, and PUFA was similar (HFD vs. HCD). A summary of all findings is in Figure 5. 

### 4.2. Expression of Inflammatory Genes 

Neuroinflammation is characterized by increased expression of inflammatory genes [22,41]. There was higher (*p* < 0.05) mRNA expression of F4/80 and Itgam on day 56 in HCD, that are markers of microglia infiltration [42,43]. The IMI was increased (46%) in HFD brain mainly because IL-10 was poorly expressed in this group (Table 3). 

## 5. Conclusions

The proportion of fat and carbohydrates in the diet modulated the speed of deposition of lipids and composition of brain FA. These changes were associated with the expression of inflammatory genes.

## Figures and Tables

**Table 1:** Primer sequences of real time PCR.

Gene	RefSeq	Sense	Antisense
Cox 2	YP_001686701.1	AACATCCCCTT CCTGCGAAG	AAGTCC ACTCCATGGCCCAG
F4/80	NM_010130.4	CCTGAACATGCAACCTGCCAC	GGGCATGAGCAGBCTGTAGGATC
iNOS	NM_001313921.1	CGGCAAACCCAAGGTCTACG	CACCTGCTCCTCGCTCAAGTTC
Il-6	NM_001314054.1	GGTAGCATCCATCATTTCTTTG	CGGAGAGGAGACTTCACAAGAG
Il-1β	NM_008361.4	GGCAGCTACCTGTGTCTTTCCC	ATATGGGTCCG ACAGCACGAG
TNF-α	NM_001278601.1	TCTTCTCATTCCTGCTTGTGGC	CACTTGGTGGTTTGCTACGACG
Il-10	NM_010548.2	TGCCAAGCCTTATCGGAAATG	AAATCGATGACAGCGCCTCAG
Itgam	NM_001082960.1	TAATGACTCTGCGTTTGCCCTG	ATTGGAGCTGCCCACAATGAG
Aif1	NM_019467.2	CCTGAGGAGATTTCAACAGAAGC	GGACCGTTCTCACACTTCCC

Abbreviations: Cox 2—cyclooxygenase-2; iNOS—inducible nitric oxide synthase; Il—interleukin; TNF-α—tumor necrosis factor alpha; Itgam—integrin subunit alpha m; Aif1—allograft inflammatory factor 1.

**Table 2:** Fatty acid family composition (mg/100 g of sample) and n-6/n-3 fatty acid, PUFA/SFA, and MUFA/SFA ratios in the brains of mice fed a high carbohydrate diet (HCD) or high fat diet (HFD) before (day 0) and 7, 14, 28, or 56 days after onset of diet administration. Key: ∆%: percentage of change.

Fatty Acids		0 (Day)	7 (Day)	14 (Day)	28 (Day)	56 (Day)	∆%
SFA	HCD	3438.6 ± 13.1	3382.6 ± 9.7	3761.6 ± 34.9 ^a,b^	5577.5 ± 47.2 ^a,b,c^	5905.9 ± 23.9 ^a,b,c,d^	71.7%
	HFD		4363.6 ± 15.0 ^a,^*	6157.7 ± 41.7 ^a,b,^*	6047.2 ± 26.0 ^a,b,c,^*	6122.4 ± 34.4 ^a,b,c,d,^*	78.0%
MUFA	HCD	1709.0 ± 41.6	1632.3 ± 17.3	2147.9 ± 101.9 ^a,b^	2956.7 ± 119.1 ^a,b,c^	3202.5 ± 31.1^a,b,c,d^	87.3%
	HFD		2072.2 ± 29.7 ^a,^*	2859.4 ± 36.2 ^a,b,^*	3012.9 ± 22.8 ^a,b,c^	3035.1 ± 24.8 ^a,b,c,^*	77.5%
PUFA	HCD	2665.6 ± 26.8	2660.1 ± 21.5	2938.0 ± 60.4 ^a,b^	4245.7 ± 57.2 ^a,b,c^	4504.6 ± 36.8 ^a,b,c,d^	68.9%
	HFD		3401.8 ± 31.1^a,^*	4784.2 ± 25.9 ^a,b,^*	4693.0 ± 11.5 ^a,b,c,^*	4810.7 ± 9.0 ^a,b,d,^*	80.4%
*n*-6	HCD	1224.8 ± 23.1	1189.1 ± 18.3	1360.3 ± 29.8 ^a,b^	1914.9 ± 20.8 ^a,b,c^	2094.9 ± 8.5 ^a,b,c,d^	71.0%
	HFD		1574.3 ± 13.2 ^a,^*	2182.3 ± 22.9 ^a,b,^*	2141.6 ± 8.0 ^a,b,c,^*	2192.5 ± 8.0 ^a,b,d,^*	79.0%
*n*-3	HCD	1440.8 ± 13.5	1471.0 ± 11.3	1577.7 ± 52.5 ^a,b^	2330.8 ± 53.3 ^a,b,c^	2409.8 ± 35.8 ^a,b,c^	67.2%
	HFD		1827.5 ± 28.2 ^a,^*	2601.9 ± 11.9 ^a,b,^*	2551.4 ± 8.2 ^a,b,c,^*	2618.2 ± 4.1 ^a,b,d,^*	81.7%
PUFA/SFA	HCD	0.78 ± 0.01	0.79 ± 0.01	0.78 ± 0.02	0.76 ± 0.02	0.76 ± 0.01	−2.6%
	HFD		0.78 ± 0.01	0.78 ± 0.01	0.78 ± 0.01	0.79 ± 0.01	1.2%
MUFA/SFA	HCD	0.50 ± 0.03	0.48 ± 0.01	0.57 ± 0.05 ^a,b^	0.53 ± 0.04	0.54 ± 0.01 ^b^	8.0%
	HFD		0.47 ± 0.02	0.46 ± 0.02 ^a^	0.50 ± 0.01^b^	0.50 ± 0.01 ^b,c^	0.0%
*n*-6/*n*-3	HCD	0.85 ± 0.02	0.81 ± 0.02 ^a^	0.86 ± 0.04 ^b^	0.82 ± 0.03 ^a,c^	0.87 ± 0.02 ^b,d^	2.3%
	HFD		0.86 ± 0.02	0.84 ± 0.01 ^b^	0.84 ± 0.01 ^b^	0.84 ± 0.01 ^b^	−1.1%
SUM	HCD	7813.3	7675.1	8847.5	12792.5	13613.1	74.2%
	HFD		9837.7	13801.3	13753.1	13968.2	78.7%

Results expressed as the mean ± standard deviation of three replicates for each group. Abbreviations: SFA: total saturated fatty acids, MUFA: total monounsaturated fatty acids, PUFA: total polyunsaturated fatty acids, SUM: sum of all fatty acids evaluated. HCD: High-carbohydrate diet, HFD: High fat diet. *p* < 0.05 as compared with day 0 (**a**), day 7 (**b**), day 14 (**c**), and day 28 (**d**), and HCD group*.

**Table 3:** mRNA expressions (arbitrary units) of inflammatory genes in the brains of mice fed with either high carbohydrate diet (HCD group) or high fat diet (HFD group) for 56 days.

Genes	Total Brain Tissue	
	HCD	HFD
F4/80	1.41 ± 0.21	0.75 ± 0.11 *
IL-6	1.12 ± 0.14	0.92 ± 0.08
IL-1β	1.52 ± 0.23	1.28 ± 0.16
TNF-α	0.49 ± 0.06	0.26 ± 0.05
iNOS	1.10 ± 0.11	1.23 ± 0.33
IL-10	2.09 ± 0.55	1.23 ± 0.33
COX-2	1.12 ± 0.11	1.33 ± 0.17
Itgam	1.31 ± 0.18	0.83 ± 0.08 *
Aif1	1.63 ± 0.22	0.89 ± 0.20
IMI	10.38	15.20

mRNA expression in total brain tissue homogenate. β2-microglobulin (β2m) was used as housekeeping gene. Results are expressed as means ± standard deviation (*n* = 8–10). HCD—high-carbohydrate diet; HFD—high-fat diet; IL—interleukin; Cox 2—cyclooxygenase 2; iNOS—inducible nitric oxide synthase; TNF-α—tumor necrosis factor alpha; Itgam—integrin subunit alpha m; Aif1—allograft inflammatory factor 1. Inflammatory marker index, i.e., IMI = (F4/80 + IL-6 + IL-1β + TNFα + iNOS + COX-2 + Itgam + Aif1)/IL-10. * *p* < 0.05 as compared with the HCD group.
